# Supplementary material for: Color discrimination and gas chromatography-mass spectrometry fingerprint based on chemometrics analysis for the quality evaluation of Schizonepetae Spica
Source: PLoS One. 2020 Jan 7;15(1):e0227235. doi: 10.1371/journal.pone.0227235 (PMC6946158; doi:10.1371/journal.pone.0227235)
Supplement: Attachment — Submitted filename: Response to Reviewers.docx [file pone.0227235.s001.docx]

Academic editor

1. The sentence “color discrimination is an important aspect for the macroscopic identification of Chinese medicinal material (CMM) and for the evaluation of quality in traditional experiences” has been revised as “color discrimination is an important aspect for the macroscopic identification of Chinese medicinal material (CMM)” as shown in section Introduction.

2. The red-green-blue (RGB) values were extracted from the images of samples using Photoshop CS6 (Adobe Systems Inc., USA) software (Table 1). The surface color of the Schizonepetae Spica calyx was selected to extract RGB values. Thank you for your kindly recommendation. Table 1 has been inserted after the Discrimination the color of Schizonepetae Spica calyx section.

Reviewer #1

1. The yellowish-green color of SS corresponded to a relatively higher concentration of volatiles (~ 0.69%-1.65% mL/g), while SS in brownish color was correlated with relatively lower volatiles concentration (~ 0.47%-0.85% mL/g) (in section Yield of essential oils from Schizonepetae Spica of different colors). In addition, pulegone, which is known for its pleasant odor, analgesia, anti-inflammatory and antiviral properties, is the chemical indicator in the Chinese Pharmacopoeia (2015 edition) of SS (in section Identification of volatile components from Schizonepetae Spica and section Partial least-squares discriminate analysis). In our manuscript, we demonstrated that the relative content of 4,5,6,7-tetrahydro-3,6-dimethyl-benzofuran and pulegone could be applied as marker components for quality evaluation of SS samples in different colors. And the relative content of pulegone was higher in the yellowish-green-type than in the brownish-type samples (in section Partial least-squares discriminate analysis). Therefore, we can draw a conclusion that SS of good quality were often yellowish-green and those of poor quality were often brownish.

2. Thank you for your kindly recommendation. The section Identification of volatile components from Schizonepetae Spica has been inserted before the section of Hierarchical clustering analysis in the manuscript.

3. In table 2, “Molecule structure” has been revised as “Molecular Formula”.
